# Attentional Differences in a Driving Hazard Perception Task in Adults with Autism Spectrum Disorders

**DOI:** 10.1007/s10803-016-2965-4

**Published:** 2016-11-19

**Authors:** Elizabeth Sheppard, Editha van Loon, Geoffrey Underwood, Danielle Ropar

**Affiliations:** School of Psychology, University of Nottingham, University Park, Nottingham, NG7 2RD UK

**Keywords:** Attention, Autism spectrum disorders, Driving, Eye-tracking, Hazard perception

## Abstract

The current study explored attentional processing of social and non-social stimuli in ASD within the context of a driving hazard perception task. Participants watched videos of road scenes and detected hazards while their eye movements were recorded. Although individuals with ASD demonstrated relatively good detection of driving hazards, they were slower to orient to hazards. Greater attentional capture in the time preceding the hazards’ onset was associated with lower verbal IQ. The findings suggest that individuals with ASD may distribute and direct their attention differently when identifying driving hazards.

## Introduction

Driving a car is an important life skill that allows increased mobility and independence, along with associated physical, social and economical benefits (Collia et al. 2003). However survey data have reported that only around 25% of adults with Autism Spectrum Disorders (ASD) drive independently in comparison with 75% of the population as a whole, suggesting that it might be a difficult task for people with ASD to master (Feeley 2010). ASD individuals who do drive rate their own driving ability as poorer than typically developing individuals, and are more likely to report being in accidents where they hit another car or person, and engaging in mistakes, lapses and intentional violations while driving (Daly et al. 2014).

Simulator studies suggest that individuals with ASD may find a range of specific skills involved in driving to be challenging. For instance, Classen et al. (2013) reported poorer performance in a number of aspects of simulator driving in ASD, including operation skills, lane maintenance, and speed regulation. Cox et al. (2016) also report poorer simulator driving in a cohort of individuals with ASD, which was further impaired by a concurrent working memory task. Reimer et al. (2013) measured eye movements during a simulator drive and found that participants with ASD tended to look further into the distance than comparison individuals, which the authors suggested could be less useful for detecting rapidly changing situations on the roads. Moreover, when placed under increased attentional demands the participants with ASD tended to shift attention away from the roadway, which might affect their ability to detect (and respond to) hazardous events safely.

Hazard perception (HP), the ability to identify and respond to potentially dangerous events on the roads, has been previously shown to pose some difficulties for those with ASD (Sheppard et al. 2010). HP proficiency is measured by presenting participants with videos filmed from the point of view of a driver of a car travelling down a road and asking them to identify a developing hazard by pressing a button on a keyboard. As experienced drivers typically respond faster than novices (e.g. Scialfa et al. 2011; Wetton et al. 2011), and HP test scores predict crash risk (e.g. Boufous et al. 2011; Horswill et al. 2015), HP ability is believed to be an important component of driving.

Individuals with ASD were found to be less accurate than typically developing individuals in detecting hazards, primarily due to poorer performance when the hazard arose from the actions of a clearly visible person (social hazards) rather than when the source of the hazard was obscured (non-social hazards, such as another car driver; Sheppard et al. 2010). These results are consistent with previous research that has found reduced attention to social stimuli in individuals with ASD. For instance, individuals with ASD spend less time attending to people (Klin et al. 2002) and are slower to fixate their attention on people in scenes (Freeth et al. 2010; Fletcher-Watson et al. 2009). However, Sheppard et al. also found participants with ASD were slower to detect hazards—both social and non-social—than typically developing comparison individuals. This suggests that individuals with ASD may experience some general difficulties with HP beyond any specific problem with hazards involving visible people. If individuals with ASD do have difficulty apprehending hazards, this could result in an elevated risk of accident involvement both in drivers and pedestrians with ASD, who need to be able to appraise the intended actions of other road users effectively. Also, as several countries (UK, Netherlands, parts of Australia) require learner drivers to pass a HP test in order to acquire their license, difficulties in doing so could represent a significant barrier to individuals with ASD gaining their license.

Although the previous research (Sheppard et al. 2010) suggested individuals with ASD have some difficulty with HP performance, it did not tell us why. Specifically, it was not clear whether those with ASD were slower and less accurate due to problems with orienting to the hazards, or whether they did attend to the hazards but were slower to appreciate their significance. Moreover, slower reaction times to hazards could reflect slowness in planning and executing responses. In the study presented here, eye movements were recorded to explore attentional patterns during a hazard perception task in individuals with and without ASD. By looking at *if* and *when* participants first fixated the hazards we could assess whether any difficulties in responding to hazards were associated with delays in or failure to orient to them. In addition, previous research has noted increased fixation durations (Chapman and Underwood 1998a) and a reduction in the spread of visual search (Underwood et al. 2011; Chapman and Underwood 1998b) around the time of the hazard occurring, which reflect attentional capture due to the impending danger. If participants with ASD fail to appreciate the danger associated with developing hazards then we would expect to see reduced or absent changes in fixation duration and spread of search at around the time of hazard onset. For all of these measures, social (i.e. hazards arising from the actions of a visible person) and non-social hazards (i.e. hazards arising from the actions of an obscured person e.g. a car) were compared to determine whether difficulties were more pronounced for social stimuli. All participants in the study completed the Autism Spectrum Quotient (AQ; Baron-Cohen et al. 2001) as a measure of the amount of autistic traits they had and these scores were related to performance on the above measures.

## Methods

### Participants

Eighteen males with ASD were recruited from specialist colleges in the West Midlands and East Midlands of England. They had all received a formal diagnosis of autism (*N* = 8) or Asperger Syndrome (*N* = 10) by a psychiatrist/clinical psychologist employed by the National Health Service, using DSM-IV, (American Psychiatric Association, 1994). The comparison group (all male, *N* = 17) was recruited from local colleges. Details of both groups are shown in Table 1.

**Table 1:** Mean (SD in brackets) and range of age, verbal IQ, performance IQ, full-scale IQ, and Autism Spectrum Quotient score for the ASD and comparison groups

	Age	VIQ	PIQ	FSIQ	AQ
ASD (N = 18)	18.79 (2.08) 17.17–24.92	85.94 (16.39) 64–125	91.06 (19.65) 58–127	87.11 (18.66) 60–124	23.72 (6.14) 12–33
Comparison (N = 17)	18.19 (1.43) 16.42–21.08	95.12 (8.47) 85–110	93.29 (9.57) 76–109	93.47 (8.03) 78–109	17.06 (6.31) 8–29

The Wechsler Abbreviated Scale of Intelligence (WASI) was carried out on all participants to establish levels of verbal and non-verbal ability. The groups did not differ significantly in chronological age, performance or full-scale IQ. However, the difference between groups in verbal IQ did reach significance, *t*(33) = 2.10, *p* = .046. Hence, verbal IQ was entered as a covariate where possible in analyses. All participants were attending colleges for over 16-year-olds full-time. While some participants in both groups were studying for A-levels, the majority were completing vocational courses (such as National Diplomas, NVQs etc.) All participants completed the Autism Spectrum Quotient (Baron-Cohen et al. 2001) to gain an indication of the strength of autistic traits in both groups. The group with ASD had significantly higher AQ scores than the comparison group *t*(33) = 3.17, *p* < .005.

As in Sheppard et al. (2010), non-drivers were recruited in order to assess ability to detect hazards prior to any training or the development of specific HP strategies. As it has been shown that HP performance improves with driving experience (e.g. Wetton et al. 2011) the recruitment of non-drivers also controlled for level of experience. However, all participants were regular passengers in motor vehicles. Individuals were also excluded if they had any known visual or motor impairment or other comorbid diagnoses. All participants were tested in a familiar setting within their college.

### Design

A mixed 2 × 2 design was employed whereby both groups (ASD and Comparison) viewed all 20 hazard videos (10 social and 10 non-social). The videos were displayed in one of two fixed sequences: the first was randomly generated and the second was the reverse order.

In order to assess eye movement parameters during periods of interest in relation to the hazards occurring, the videoclips were divided into three consecutive pre-defined time windows, which will be referred to as “outside”, “precursor” and “hazard”. These time windows were defined based on previous research (e.g. Underwood et al. 2011). The outside window started at the beginning of the videoclip and ended at the start of the precursor window. The precursor window commenced when the hazardous road user was visible and acting in such a way to indicate that a hazard may occur but had not yet reached the point at which an evasive action would be required from the driver. The hazard window immediately followed the precursor window and started at the point when the hazardous road user’s actions required evasive action by the driver. For example, imagine a situation where a pedestrian is walking along the pavement, she turns, strolls to the side of the road then steps out into the road. The outside window covers the time up until the pedestrian turns towards the roadside (and any additional time before the pedestrian comes into view). As the pedestrian walks towards the edge of the pavement, she is now behaving in such a way that the hazard might be anticipated, although no evasive action is required yet: this is the precursor window. The point at which the pedestrian steps into the road is the end of the precursor window and the start of the hazard window (hazard onset), which continues up until the participant responds. The outside window ranged from 1.11 to 11.07 s with a mean length of 5.05 s (*SD* = 2.99). The source of the hazard was sometimes present from the start of the outside window but in other cases came into view during the outside window. In some clips (two social and one non-social), where the hazard onset abruptly, the source of the hazard was not present during the outside window at all and there was no precursor window. For the remaining 17 clips, the precursor window ranged from 0.36 to 10.51 s with a mean length of 4.51 s (*SD* = 3.52). The hazard window ranged from 0.83 to 10.45 s with a mean length of 4.00 s (*SD* = 2.54).

The eye-tracking analysis focused on when the source of the hazard was first fixated, as well as the mean fixation durations and horizontal spread of fixations during the three time windows of viewing: outside, precursor and hazard. The groups were also compared on two behavioural measures: accuracy (number of hazards correctly identified) and reaction time (time from the start of the precursor window until a correct response was made).

### Materials and Apparatus

All participants were presented with 20 videos of driving situations, recorded from the viewpoint of the driver. Although all clips contained a mixture of cars, pedestrians and sometimes other vehicles, each video contained only one hazardous driving event. In ten of the videos the source of hazard was a clearly visible person (i.e. pedestrian)—referred to as ‘social hazards’—while in the other ten, the source of the hazard was a vehicle wherein the driver was obscured from sight—referred to as ‘non-social hazards’. This distinction between social and non-social hazards was used in Sheppard et al. (2010) and based on research that suggests that people conceive of vehicles such as cars as non-social entities even though they are controlled by a person (Walker 2005), while other road users with a visible human figure (e.g. cyclists, pedestrians) engage socio-cognitive processing. The social hazard clips involved: children playing football, which was kicked into the road and chased after by the children; people crossing at a pedestrian crossing (two clips); a man who jogs across the road to meet his friend who waved from the other side; a man who rushes across the road to the bus stop; a group of youths who saunter out into the road; a group of school children, one of whom is pushed into the road; a man who wanders into the road while looking in the wrong direction; a man who sprints across the road; a jogger who is using the road for running. The non-social hazard clips were as follows: car reversing out of a driveway or parking space into the road (two clips); van/car pulling out from a side road (three clips); car changing lanes abruptly in front of observer (two clips); car pulling into the observer’s lane to move around parked vehicles (three clips).

Eye movements were recorded using a remote Tobii 1750 eye-tracker system (recording frequency of 50 Hz and accuracy of 1° of visual angle). The images were displayed on a 19″ colour LCD monitor at a distance of approximately 60 cm and subtended a visual angle of approximately 32° horizontally and 24° vertically. The screen resolution was set to 1024 × 768 pixels. Participants were free to move their head throughout the experiment but were asked to ‘‘sit quite still’’. A 5-point calibration was conducted using Clearview software.

### Procedure

Participants were informed that they would view video clips in which they needed to identify driving hazards. They were asked to press the space bar on the keyboard as quickly as possible when they saw a hazard developing. A definition of a driving hazard was provided, as used in previous studies (e.g. Crundall et al. 2002; Sheppard et al. 2010; Underwood et al. 2013), to ensure that participants understood the task. They were told that a hazard was ‘an event that occurs on the road whilst you are driving along that would make you have to consider taking some kind of action to avoid an accident’. Participants were told that each video may contain one or more driving hazards, or may not contain any. Although each video actually contained only one hazard, participants were not informed that this was the case. No feedback was given in regards to accuracy on each trial.

Each video was preceded by a fixation point displayed for 1 s. When participants made a key-press the clip terminated showing a blank screen, and response time was recorded. At this point the participants were required to report the hazard to the experimenter. The experimenter asked “what was the hazard?” and wrote down the participants’ verbal responses. If the participant made a response that was ambiguous, such as “it was the car” when more than one car was present in the scene, the experimenter probed him further to obtain a clear description of the source and nature of the hazard by saying “Can you be more precise?” The experimenter then pressed a key to move to the next video.

### Measures and Statistical Methods

For the eye-tracking analyses some participants were excluded due to a large amount of missing data (≥45%). As a consequence the analyses were carried out with 15 participants with ASD and 16 comparison participants. These two groups did not differ significantly in the mean amount of missing eye-tracking data, (ASD *M* = 10.44, *SD* = 8.13; Comparison *M* = 10.45, *SD* = 11.00), t(29) = 0.004, *p* > .99. The eye movements were overlaid on the videoclips (using the raw co-ordinates of gaze position at each sampling point) and were handcoded frame-by-frame by one of the researchers to determine at what time after the start of the precursor window the hazard was first fixated by the participant. Due to variability in the latencies to first fixation of the hazards, they were converted to z-scores for each clip and mean z-scores were used in subsequent analyses.

Responses were regarded as correct if the participant made a key-press *and* gave a correct verbal explanation. Hazard reaction times were analysed for correct responses only, from the start of the precursor window until a key-press was made. As reaction times varied greatly between clips, mean z-scores of reaction times were calculated. Subsequently, a transformation was carried out to normalise the data: √(z-score + 2) prior to analysis.

A measure of the speed of reaction was calculated by subtracting the latency to first fixation of the hazard from the hazard reaction time for each participant for each clip, essentially giving an indication of how long after the participant looked at the hazard they made their key press response. Again, these reaction speeds varied between clips so data were transformed to z-scores for each of the 20 clips.

The above measures were analysed using 2 × 2 ANOVA with group (ASD or comparison) as a between-participants factor and hazard type (social or non-social) as a within-participants factor. As VIQ differed significantly between the groups, the analyses were repeated with VIQ entered as a covariate.

Attentional capture was measured by calculating the mean fixation duration and mean horizontal spread of fixations (standard deviation of the horizontal (x) coordinates of fixations in pixels) for each of the three pre-defined time windows (outside, precursor, and hazard). Fixations were extracted using the ClearView fixation filter (default settings), which deems individual gaze samples within a distance of 30 pixels as being part of the same fixation, with a minimum temporal duration of 100 ms. Matlab was used to extract the above metrics for the three time windows. These were analysed with 3 × 2 × 2 ANOVA with timing (outside or precursor or hazard) and type of hazard (social or non-social) as within-participants factors and group (ASD or comparison) as a between-participants factor. The analyses were also repeated with VIQ as a covariate.

Although eye movements were the main focus of this study, behavioural analyses of accuracy and reaction time were also carried out, using data from all participants. Mann–Whitney tests were used to compare the number of social and non-social hazards participants in the two groups correctly identified. Correlation analyses were used to determine the extent to which the variables measured in this study relate to levels of autistic traits and verbal IQ, as well as to determine the relationship between latency to first fixation of the hazard and hazard reaction time.

## Results

### Eye Movements

Analysis of basic eye-tracking measures in the period prior to the appearance of the source of the hazard (i.e. the outside window) did not reveal any group differences in number of fixations, fixation duration, or horizontal spread of search. This suggests that individuals with ASD did not have any notable differences in their general scanning or attention towards the scene before the hazard started to occur. There was also no significant difference between the groups in the mean total fixation time (ASD *M* = 8.14 s vs. Comparison *M* = 8.27 s).

All participants fixated the source of the hazard in each clip at some point after its appearance on the screen, typically during the precursor window. Mean times for first fixations of social and non-social hazards are displayed in Table 2 (although z-scores were used in analysis).

**Table 2:** Mean time to first fixate and speed of reaction to social and non-social hazards in ms (SD in brackets)

	First fixation social	First fixation non-social	Reaction speed social	Reaction speed non-social
ASD (N = 15)	2103.99 (616.14)	1876.12 (199.85)	2711.57 (759.71)	3657.37 (1010.83)
Comparison (N = 16)	1797.08 (589.64)	1464.74 (115.98)	3186.60 (786.69)	3630.03 (1022.71)

**Fig. 1:**
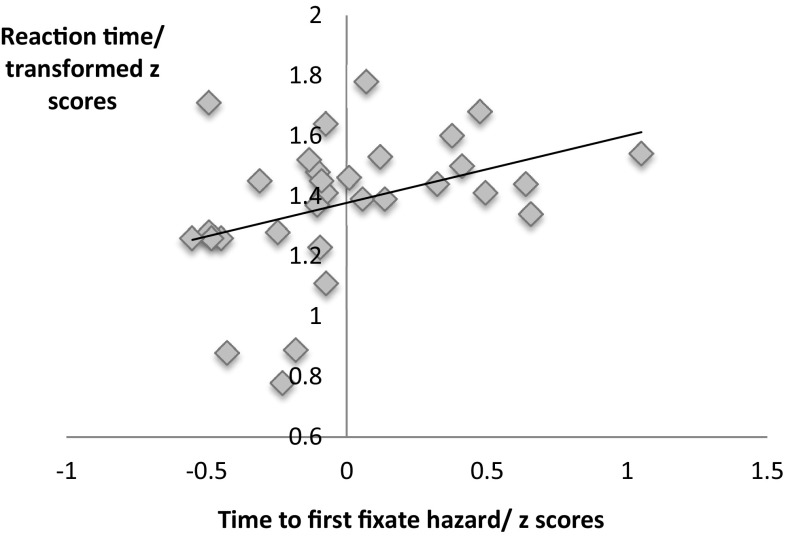
Hazard reaction time plotted against time to first fixate the hazard (both plotted as z-scores), with regression line

Participants with ASD were slower to first fixate the hazardous target (*M* = 0.22, *SD* = 0.41) than comparison participants (*M* = −0.19, *SD* = 0.25), *F*(1,29) = 11.05, *p* < .005, η_p_^2^ = 0.27. There was no effect of hazard type or interaction between hazard type and group. Entering VIQ as a covariate did not alter this pattern of results. There was a significant correlation between time to first fixation and hazard reaction time, r(29) = 0.38, *p* < .05, depicted in Fig. 1, indicating that participants who took longer to first look at the hazards also responded to them later. Table 2 also displays the mean reaction speed i.e. time between first looking at the hazard and making a key-press response. There were no group differences on this measure suggesting that there were no differences between the groups in the time they took to respond to the hazard having oriented to it.

**Fig. 2:**
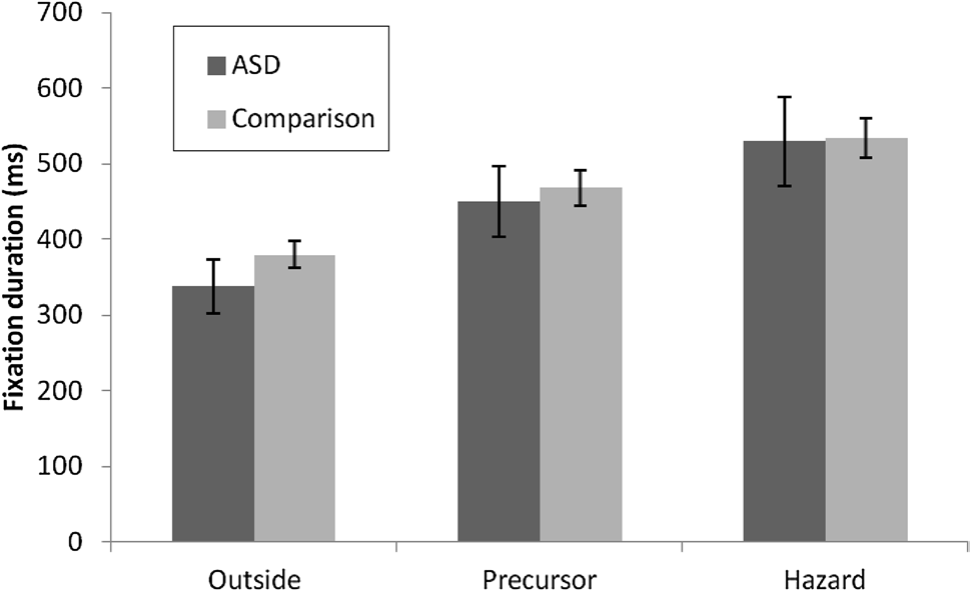
Mean fixation durations (in ms) for individuals with and without ASD in outside, precursor and hazard time windows (*error bars* show the standard error)

Previous research has shown that fixation durations tend to increase around the point of hazard onset, while the spread of fixations in the horizontal plane decreases around the same time suggesting attentional capture (e.g. Chapman and Underwood 1998a, b; Underwood et al. 2005). As seen in Fig. 2 there is a linear increase in mean fixation durations across the three time windows in the approach to hazard occurrence for both groups.

There was an effect of time window on mean fixation duration, *F*(2, 56) = 80.95, *p* < .001, η_p_^2^ = 0.74 Post-hoc pairwise comparisons (Bonferroni corrected) revealed that mean fixation durations were longer for the precursor than the outside windows, and longer for the hazard windows than the precursor and outside windows (all *p* < .001). There was no effect of group (ASD or comparison) or any interactions. Entering VIQ as a covariate did not alter this pattern of results.

**Fig. 3:**
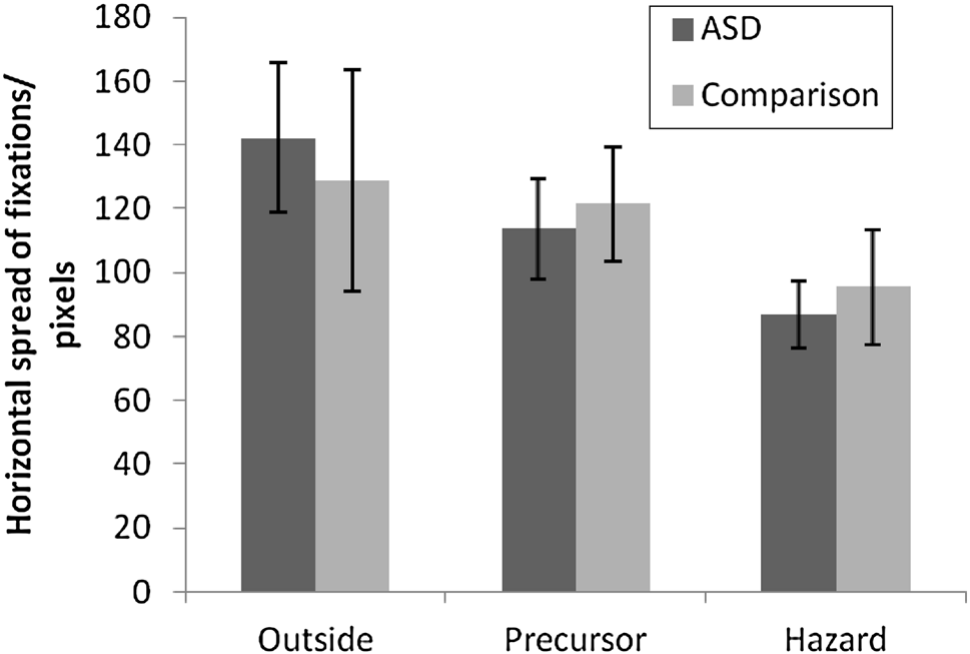
Mean horizontal spread of fixations (in pixels) for individuals with and without ASD in outside, precursor and hazard time windows (*error bars* show the standard error)

The horizontal spread of fixations across all hazard types in the three time windows is displayed in Fig. 3. There was a main effect of time window on spread of fixations, *F*(2,58) = 49.82, *p* < .001, η_p_^2^ = 0.63. Post-hoc pairwise comparisons (Bonferroni corrected) revealed that the spread of fixations was greater for the outside than the precursor window (*p* < .005), for the outside than the hazard window, and for the precursor than the hazard window (both *p* < .001). There was a main effect of hazard type, *F*(1,29) = 4.61, *p* < .05, η_p_^2^ = 0.14, where spread of fixations was greater for clips with social hazards (*M* = 121.16, *SD* = 17.41) than clips with non-social hazards (*M* = 112.76, *SD* = 20.25). There was also an interaction between window and group, *F*(2,58) = 4.08, *p* < .05, η_p_^2^ = 0.12. Further analyses showed that the source of the interaction was differences in the reduction in spread of fixations across time windows within each group. While the ASD group showed a reduction in spread of fixations between both the outside and precursor, and the precursor and hazard windows (all *p* < .005), the comparison group only showed a reduction in spread of fixations between the precursor and the hazard windows (*p* < .005). Similar results were observed with VIQ entered as covariate apart from the interaction between time window and group was no longer significant, *F*(2,56) = 2.05, *p* = .138, η_p_^2^ = 0.07 suggesting that the above mentioned interaction may partly be accounted for by group differences in verbal IQ.

### Behavioural

All participants gave reasonable descriptions of the hazards they detected, in terms of the source and nature of the hazardous event. There was no difference between the groups in the number of social (*Mdn*: ASD = 8, Comparison = 8) or non-social hazards (*Mdn*: ASD = 8, Comparison = 9) correctly identified or for both types combined (*Mdn*: ASD = 17, Comparison = 18). For hazard reaction time there were no main effects or interactions, although the difference between groups approached significance, *F*(1,33) = 2.84, *p* = .101, η_p_^2^ = 0.079, indicating a trend towards those with ASD (*M* = 0.26, *SD* = 0.90) responding more slowly than comparison participants (*M* = −0.16, *SD* = 0.70). Including VIQ as a covariate did not alter his pattern of results.

### Relationships with AQ Scores and IQ

The AQ was used in this study to gain an indication of the amount of autistic traits the participants had, both those with a pre-existing diagnosis of ASD and the comparison participants. Measures of IQ were taken as an indication of the cognitive ability levels of the groups. Table 3 displays correlations between the various eye-tracking and behavioural measures presented in the previous sections, AQ and IQ. There was a significant correlation between AQ scores and time to first fixate the hazard (z-scores) indicating that individuals higher in autistic traits tended to look at the source of the hazard later than those lower in autistic traits. AQ scores also correlated significantly with (transformed z-scores) hazard reaction time, suggesting that those higher in autistic traits tended to respond later to hazards than those with lower AQ scores. AQ scores showed a marginal negative correlation with hazard perception accuracy, indicating that individuals with higher levels of autistic traits tended to correctly identify fewer hazards than those lower in autistic traits. None of these measures correlated significantly with any of the IQ indices.

**Table 3:** Correlations between eye-tracking and behavioural measures, Autism Spectrum Quotient (AQ) and IQ scores

	AQ	VIQ	PIQ	FSIQ
Latency to fixate hazard	0.545**	−0.233	−0.207	−0.256
Hazard reaction time	0.409*	0.036	0.114	0.081
Reaction speed	0.040	0.085	0.068	0.017
Hazard perception accuracy	−0.333^a^	0.317	0.304	0.306
Change in spread of fixations (outside to precursor)	0.103	−0.487**	−0.003	−0.247
Change in spread of fixations (outside to hazard)	0.011	−0.257	0.225	0.013
Change in fixation duration (outside to precursor)	−0.061	0.234	0.154	0.212
Change in fixation duration (outside to hazard)	−0.153	0.121	−0.024	0.040

All correlations are Pearson correlations apart from Hazard perception accuracy which used Spearman’s Rho

*Significant at 0.05 level

**Significant at 0.01 level

^a^Marginally significant (p = .053)

For each participant the mean spread of fixations during the precursor window was subtracted from the mean spread of fixations during the outside window, and similarly, the spread of fixations during the hazard window was subtracted from the mean spread of fixations during the outside window. Likewise for each participant the mean fixation duration in the outside window was subtracted from the mean fixation duration in (a) the precursor window and (b) the hazard window. This yielded variables that reflect the change in fixation duration and spread of search occurring across the course of the videos. These variables did not relate to AQ scores but there was a significant negative correlation between verbal IQ and the change in horizontal spread of fixations between the hazard and precursor window. Individuals with lower verbal ability tended to show a greater narrowing in spread of search during the precursor window than those of higher verbal IQ.

## Discussion

This study provided evidence of atypical attentional processing of driving hazards in individuals with ASD. Individuals with ASD did look at the relevant parts of the scene; however comparison participants fixated the source of the hazard earlier than the ASD group. In addition, the time taken to look at the source of the hazard once it had started to develop correlated with AQ scores, suggesting that those who have more autistic traits (whether or not they have a diagnosis of ASD) are slower to orient to driving hazards. There were no accuracy or reaction time differences between social and non-social hazards, suggesting these differences may not be specific to social stimuli. Importantly, the time that elapsed between orienting visually to the hazards and responding with a key-press did not differ between groups, and did not relate to AQ scores. This suggests that once a hazard was fixated on it was responded to just as quickly. Together these findings suggest that slowness in responding to hazards in individuals with ASD or with autistic traits may relate more to a failure to orient to them early than to not understanding their significance or difficulties in formulating a response.

As longer times in fixating the source of the hazard were not restricted to social hazards, a general processing explanation is needed. One possibility is that the ASD group found it more difficult to shift their attention between different items in the scene (Courchesne et al. 1994; Leekam and Moore 2001) but this seems unlikely as the groups did not differ on number of fixations, spread of horizontal search, or fixation duration during the outside window, prior to any hazard developing. Instead the results imply that participants with ASD had poorer search strategies. They may be less inclined to focus their attention on the components of the scene which are most likely to be the source of a hazard, perhaps due to having a poorer understanding of where a hazard might appear within a given environment. This is consistent with findings of Reimer et al. (2013), who measured eye movements during a simulator drive. They report that participants with ASD tended to focus on sub-optimal parts of the environment, which might prevent them from gaining an early indication of rapidly changing situations on the roadway, including driving hazards. Another possibility is that the slowness to orient to hazards is due to a failure to prioritise attention to social information in a scene in individuals with ASD. Although the hazards involving other cars did not contain a visible human, the cars are nevertheless controlled by other humans and thus could be construed as social agents. Previous research suggests that typical adults readily attribute mental states to non-human agents, but people with ASD may be less inclined to do so (Castelli et al. 2002). Hence, it may be that participants with ASD were slower to orient to both hazard types because they are social in nature. On the other hand, previous research suggests that hazards that do contain a visible human are conceived of differently from those that do not (Walker 2005) so the status of the non-social hazards is unclear.

Despite their delay in orienting to the hazard, participants with ASD showed the typical increase in fixation duration during the precursor and hazard phases of the video clips consistent with previous studies with typically developing drivers (Underwood et al. 2005). An increase in fixation duration in the approach to a hazardous event implies attentional capture. Consequently, it can be assumed that although those with ASD oriented to hazards slightly later, once they had done so they did perceive the imminent danger of the situation even within the precursor window. Therefore, there was no support for the suggestion that those with ASD did not apprehend the events taking place as dangerous.

An exploration of spread of search across different time windows of the hazard revealed distinct differences between groups. Individuals with ASD showed a significant reduction in their horizontal spread of search between the outside and precursor stages, which was not observed in comparison participants. Further analysis suggested that this narrowing spread of visual search during the precursor window in those with ASD may be accounted for by the difference between the groups in verbal IQ. Verbal IQ correlated negatively with the change in spread of search between the outside window (where the hazard had not yet started to develop) and the precursor window (where the hazardous event could be anticipated but had not yet taken place), while AQ scores were unrelated with this measure. Thus, the findings suggest that while slowness in orienting to (and subsequently identifying hazards) may be associated with having a diagnosis of ASD or autistic traits, differences in spread of attention may be associated with lower levels of verbal ability rather than an ASD diagnosis or having autistic traits.

This narrowing spread of search may reflect greater attentional capture in those with lower verbal IQ. It is possible that those with lower IQ have fewer attentional resources (Hunt 1980) and so once they identify a potential hazard their attention is fully utilised processing it and they are unable to maintain a broader search. A wider field of visual search may be strategic as it allows one to pick up on important information in the periphery, which can be critical in determining if the potential hazard is likely to actually transpire (Crundall et al. 1999). Consistent with this, experienced drivers direct their attention broadly to parts of the environment that are most informative about potential dangers, whereas novice drivers tend to maintain a smaller field of visual search perhaps also because of insufficient attentional resources (Underwood 2007). However, while it is reasonable to suggest that those with lower IQ may have more limited attentional resources, it is not clear why the narrowing spread of search related specifically to verbal IQ and not performance IQ.

The lack of group difference in accuracy in detecting hazards contrasts with a previous study that did observe differences in accuracy between groups with and without ASD (Sheppard et al. 2010), although in the current study overall accuracy did correlate negatively with AQ scores. This discrepancy may be due to differences in the video clips presented. In Sheppard et al.’s study (2010) participants were not limited in the number of key-presses they could make per clip whereas in the current study, the eye-tracking programme terminated the clip once a key-press was made. Therefore, to minimise false alarms, clips with only one clear instance of a hazard were selected which may have resulted in ceiling effects with respect to accuracy. This possibility appears to be supported by the observation of fewer key-presses to non-hazardous events in this than in the previous study.

While similarity in behavioural performance in this study appears promising for driver safety in those with ASD, the subtle attentional differences observed in those with ASD in the current study might lead to poorer hazard detection in situations where demands are higher due to there being a number of potential hazards competing for attention, or where stress levels are higher due to the other simultaneous activities involved in real-life driving and the greater risks to the driver associated with making an error. Therefore, future research should also explore hazard perception under high and low attentional loads, and conditions closer to those experienced on the roads. Nevertheless, a video-based hazard perception test similar to the one used in this study forms part of driver licensing in several countries—therefore, it is important to understand the HP test performance of those with ASD as if they have difficulties this could present an obstacle to acquiring a driving license.

Finally, it is worth bearing in mind some limitations of the current research. Firstly, all participants were young adults who had not yet acquired their license. This population was selected to be consistent with the previous study (Sheppard et al. 2010) and also to provide a baseline indication of ability in HP prior to any specific training or experience that could influence this skill. It is important to be aware that HP test performance in this study is unlikely to reflect performance that would be observed after training/practical driving experience for either group, and it would therefore be a priority to investigate these skills in experienced drivers with ASD in future. Another limitation is that this study only recruited males. Although there is no particular reason to predict that task performance would differ for females, future research could include females with and without ASD to gain a more representative sample.

Additionally, the group with ASD was signficantly lower in verbal IQ than the comparison participants, although not in performance or full scale IQ. Although the majority of aspects of HP test performance were unrelated with IQ and IQ was covaried in analyses, there was some indication that changes in the spread of attention may relate to verbal ability. Hence future research should aim to clarify how IQ impacts attention while driving in both typical and atypical populations. A further limitation is that information about whether participants were medicated was not recorded in this study, and there is evidence to suggest that medication can improve driving performance in individuals with ADHD (Martin et al. 2008).

In sum, differences between individuals with and without ASD were found in visual attentional processes deployed in a hazard perception task. Although behavioural measures (accuracy and reaction time) revealed generally comparable HP performance, eye-tracking indicated that participants with ASD were slower to first fixate hazards regardless of whether they were social or non-social. Those who were slower to fixate the hazards also tended to respond to them later, suggesting that slower HP test performance in ASD may be associated with a difficulties in orienting to hazards. Once they had oriented to the hazards, those with ASD showed typical attentional capture as evidenced by longer fixations and reduced spread of attention, implying that participants with ASD were aware of the impending danger associated with the hazards. The results also suggested that those with lower verbal IQ narrowed their spread of attention more than those of higher IQ, perhaps because they had more limited attentional resources. These subtle attentional differences should be explored further in order to understand their implications for on-road driving in those with ASD.

## References

[CR1] Ameican Psychiatric Association (1994). Statistical Manual of Mental Disorders, (DSM-IV).

[CR2] Baron-Cohen S, Wheelwright S, Skinner R, Martin J, Clubley E (2001). The Autism-Spectrum Quotient (AQ): Evidence from Asperger Syndrome/ High-functioning autism, males and females, scientists and mathematicians. Journal of Autism and Developmental Disorders.

[CR3] Boufous S, Ivers R, Senserrick T, Stevenson M (2011). Attempts at the practical on-road driving test and the hazard perception test and the risk of traffic crashes in young drivers. Traffic Injury Prevention.

[CR4] Castelli F, Frith C, Happé F, Frith U (2002). Autism, Asperger syndrome and brain mechanisms for the attribution of mental states to animated shapes. Brain: A Journal of Neurology.

[CR5] Chapman PR, Underwood G (1998). Visual search of driving situations: Danger and experience. Perception.

[CR6] Chapman PR, Underwood G, Underwood G (1998). Visual search of dynamic scenes: Event types and the role of experience in viewing driving situations. Eye guidance in reading and scene perception.

[CR7] Classen S, Monahan M, Hernandez S (2013). Indicators of simulated driving skills in adolescents with autism spectrum disorder. The Open Journal of Occupational Therapy.

[CR8] Collia DV, Sharp J, Giesbrecht L (2003). The 2001 national household travel survey: A look into the travel patterns of older Americans. Journal of Safety Research.

[CR9] Courchesne E, Townsend J, Akshoomoff NA, Saitoh O, Yeung-Courchesne R, Lincoln AJ, Lau L (1994). Impairment in shifting attention in autistic and cerebellar patients. Behavioral Neuroscience.

[CR10] Cox SM, Cox DJ, Kofler MJ, Moncrief MA, Johnson RJ, Lambert AE, Reeve RE (2016). Driving simulator performance in novice drivers with autism spectrum disorder: The role of executive functions and basic motor skills. Journal of Autism and Developmental Disorders.

[CR11] Crundall D, Underwood G, Chapman P (1999). Driving experience and functional field of view. Perception.

[CR12] Crundall D, Underwood G, Chapman P (2002). Attending to the peripheral world while driving. Applied Cognitive Psychology.

[CR13] Daly BP, Nicholls EG, Patrick KE, Brinckman DD, Schultheis MT (2014). Driving behaviors in adults with autism spectrum disorders. Journal of Autism and Developmental Disorders.

[CR14] Feeley, C. (2010). *Evaluating the transportation needs and accessibility for adults on the autism spectrum in New Jersey*. In: 89th Annual Meeting of the Transportation Research Board, Washington DC.

[CR15] Fletcher-Watson S, Leekam SR, Benson V, Frank MC, Findlay JM (2009). Eye-movements reveal attention to social information in autism spectrum disorder. Neuropsychologia.

[CR16] Freeth M, Chapman P, Ropar D, Mitchell P (2010). Do gaze cues in complex scenes capture and direct the attention of high functioning adolescents with ASD? Evidence from eye-tracking. Journal of Autism and Developmental Disorders.

[CR17] Horswill MS, Hill A, Wetton M (2015). Can a video-based hazard perception test used for driver licensing predict crash involvement?. Accident Analysis & Prevention.

[CR18] Hunt E (1980). Intelligence as an information-processing concept. British Journal of Psychology.

[CR19] Klin A, Jones W, Schultz R, Volkmar F, Cohen D (2002). Visual fixation patterns during viewing of naturalistic social situations as predictors of social competence in individuals with autism. Archives of General Psychiatry.

[CR20] Leekam SR, Moore C, Burack JA, Charman T, Yirmiya N, Zelazo PR (2001). The development of attention and joint attention in children with autism. The development of autism: Perspectives from theory and research.

[CR21] Martin L, Aring E, Landgren M, Hellström A, Andersson Grönlund M (2008). Visual fields in children with attention-deficit/hyperactivity disorder before and after treatment with stimulants. Acta Ophthalmologica.

[CR22] Reime B, Fried R, Mehler B, Joshi G, Bolfek A, Godfrey KM, Biederman J (2013). Brief report: Examining driving behavior in young adults with high functioning autism spectrum disorders: A pilot study using a driving simulation paradigm. Journal of Autism and Developmental Disorders.

[CR23] Scialfa CT, Deschênes MC, Ference J, Boone J, Horswill MS, Wetton MA (2011). A hazard perception test for novice drivers. Accident Analysis & Prevention.

[CR24] Sheppard E, Ropar D, Underwood G, van Loon E (2010). Brief report: Driving hazard perception in autism. Journal of Autism and Developmental Disorders.

[CR25] Underwood G (2007). Visual attention and the transition from novice to advanced driver. Ergonomics.

[CR26] Underwood G, Crundall D, Chapman P (2011). Driving simulator validation with hazard perception. Transportation Research F: Traffic Psychology and Behaviour.

[CR27] Underwood G, Ngai A, Underwood J (2013). Driving experience and situation awareness in hazard detection. Safety Science.

[CR28] Underwood G, Phelps N, Wright C, van Loon E, Galpin A (2005). Eye fixation scanpaths of younger and older drivers in a hazard perception task. Ophthalmic and Physiological Optics.

[CR29] Walker I (2005). Road users’ perception of other road users: Do different transport modes invoke in observers?. Advances in Transportation Studies A.

[CR30] Wetton MA, Hill A, Horswill MS (2011). The development and validation of a hazard perception test for use in driver licensing. Accident Analysis & Prevention.

